# Valuation of Lost Productivity in Caregivers: A Validation Study

**DOI:** 10.3389/fpsyg.2021.727871

**Published:** 2021-08-27

**Authors:** Aaron Gelfand, Julie Sou, Rick Sawatzky, Katrina Prescott, Alison Pearce, Aslam H. Anis, Christine Lee, Wei Zhang

**Affiliations:** ^1^School of Population and Public Health, The University of British Columbia, Vancouver, BC, Canada; ^2^Centre for Health Evaluation and Outcome Sciences, Vancouver, BC, Canada; ^3^School of Nursing, Trinity Western University, Langley, BC, Canada; ^4^Section 2 Productions, Vancouver, BC, Canada; ^5^Faculty of Medicine and Health, The University of Sydney, Sydney, NSW, Australia; ^6^Department of Pathology and Laboratory Medicine, The University of British Columbia, Vancouver, BC, Canada; ^7^Island Medical Program, University of Victoria, Victoria, BC, Canada

**Keywords:** caregiver, Valuation of Lost Productivity questionnaire, absenteeism, presenteeism, productivity loss, validity, Work Productivity and Activity Impairment questionnaire

## Abstract

**Objective:**

This study aimed to: (a) adapt the previously validated Valuation of Lost Productivity (VOLP) questionnaire for people with health problems, to a caregiver version to measure productivity losses associated with caregiving responsibilities, and (b) evaluate measurement feasibility and validity of an online version of the caregiver VOLP questionnaire.

**Methods:**

A mixed methods design was utilized. Qualitative methods, such as reviewing existing questionnaires that measured caregiver work productivity losses and performing one-on-one interviews with caregivers, were used for VOLP adaptation and online conversion. Quantitative methods were used to evaluate feasibility and validity of the online VOLP. The Work Productivity and Activity Impairment (WPAI) questionnaire for caregivers was included to compare its absenteeism and presenteeism outcomes and their correlations with VOLP outcomes.

**Results:**

When adapting the VOLP for caregivers, our qualitative analysis showed the importance of adding three major components: caregiving time, work productivity loss related to volunteer activities and caregivers’ lost job opportunities. A total of 383 caregivers who completed online survey were included in our final quantitative analysis. We found small Spearman rank correlations between VOLP and WPAI, observing a larger correlation between their absenteeism [*r* = 0.49 (95% confidence interval: 0.37–0.60)] than their presenteeism [*r* = 0.36 (0.24–0.47)]. Correlations between VOLP outcomes and total caregiving hours were larger for absenteeism [*r* = 0.38 (0.27–0.47)] than presenteeism [*r* = 0.22 (0.10–0.34)]. Correlations between WPAI outcomes and total caregiving hours were smaller for absenteeism [*r* = 0.27 (0.15–0.38)] than presenteeism [*r* = 0.35 (0.23–0.46)].

**Conclusion:**

The study provides evidence of the feasibility and preliminary validity evidence of the adapted VOLP caregiver questionnaire in measuring productivity losses due to caregiving responsibilities, when compared with the results for WPAI and the results from the previous patient-VOLP validation study.

## Introduction

Studies have consistently demonstrated that chronic conditions have a significantly negative impact on work productivity of patients (Zhang et al., 2016, 2018). However, beyond the direct impact on patients, chronic conditions such as inflammatory bowel disease, dementia, and chronic kidney disease have also been shown to have a significant impact on the work productivity of caregivers who are caring for their family members or friends who have a chronic condition (Ganapathy et al., 2015; Wang et al., 2016; Kahn et al., 2017; Fujihara et al., 2019; Kuenzig et al., 2019). For example, Fujihara et al. (2019) found that among employed family caregivers of people with dementia, the average caregiving time was 2.14 h per day. About 7.91% of their work time were missed in the past week and 35.36% of their productivity while they were working were affected. Kahn et al. (2017) found that caregivers, of a group of pediatric inflammatory bowel disease patients, had an unadjusted 214.4 ± 171.5 annual hours of work loss. This was translated to an annual lost productivity cost of $5243 USD per caregiver. Ganapathy et al. (2015) determined that caregivers of stroke patients, had a monthly mean total lost-productivity cost to be $835 USD, with 72% being attributable to presenteeism.

Many questionnaires have been developed to measure work productivity loss among people with health problems including chronic conditions (Tang et al., 2011b; Zhang et al., 2011a). Work productivity loss due to health problems commonly includes three components: (1) absenteeism (i.e., the number of days missed from work); (2) presenteeism (i.e., the reduced productivity or the productivity loss while at work); (3) employment status (change) including reduced routine work time and stopping work (Zhang et al., 2011a). For example, the Work Productivity and Activity Impairment questionnaire (WPAI) is a commonly used questionnaire to measure the impact of health problems on people’s work productivity and the Valuation of Lost Productivity questionnaire (VOLP) is a recently developed questionnaire based on economic theory to measure and value work productivity loss due to health problems in both time and monetary values (Reilly et al., 1993; Zhang et al., 2011b, 2012; Reilly Associates, 2017).

The underlying theory and concepts of work productivity loss (absenteeism and presenteeism) apply to both people with health problems and caregivers when measuring their work productivity loss. The differences of the questionnaire measuring work productivity loss among caregivers include that it needs to capture work productivity loss due to caregiving responsibilities as well as different caregiving responsibilities and the time spent on them among caregivers. Our review revealed relatively few questionnaires that have been developed, adapted or applied to measure work productivity loss due to caregiving responsibilities among caregivers. These include: (1) WPAI; (2) Work Limitations Questionnaire (WLQ); (3) iMTA Valuation of Informal Care Questionnaire (iVICQ); (4) Caregiver Indirect and Informal Care Cost Assessment Questionnaire (CIIQ) (Lerner et al., 2001, 2003, 2015; Giovannetti et al., 2009; Zhang et al., 2010; Tang et al., 2011a; Hoefman et al., 2019; Landfeldt et al., 2019). Each questionnaire has its own strengths and limitations (see details in Supplementary Appendix). In addition to incomplete components to measure caregiver responsibilities (WPAI and WLQ) and their impact on work productivity loss of caregivers (WLQ for presenteeism only), the existing questionnaires represent different approaches to measuring absenteeism and presenteeism. A 1-week recall period for absenteeism was used by WPAI and CIIQ compared to a 3-month recall period used by VOLP. Previous studies have compared and discussed the following approaches to measuring presenteeism: direct time measurement (e.g., VOLP), 0–10 scale (e.g., WPAI and CIIQ) and multidimensional measurement (e.g., WLQ) (Zhang et al., 2010, 2011a). The 0–10 scale leads to the largest time loss estimates of presenteeism when compared to direct time and multidimensional measurement methods (Zhang et al., 2010). The higher estimation might be because it captures the quality of life and psychosocial impacts as well (Zhang et al., 2010, 2011a). On the other hand, the direct time measurement provides a direct work time loss estimate that could be converted to productivity loss in monetary value. Furthermore, it is not clear whether existing questionnaires incorporated caregiver partners in their development or adaption. By including caregiver partners as research partners (i.e., patient/caregiver-oriented research), one can utilize their lived-experiences and expertise in the area potentially leading to the development of a more accurate tool that better measures caregiving responsibilities and the resulting productivity losses among caregivers.

Our objectives were to use a caregiver-oriented research approach to adapt a previously validated version of the VOLP questionnaire for people with health problems, to a caregiver version to measure productivity losses due to caregiving responsibilities, and then to develop and evaluate the feasibility and validity of an online version of the caregiver VOLP questionnaire.

## Methods

We used a mixed methods design, where qualitative methods were used for VOLP adaptation and online conversion and quantitative methods for online survey feasibility and validity testing. We defined caregivers as individuals currently caring for a family member or friend living with a chronic condition. There were some differences from the way previous studies defined caregivers (Giovannetti et al., 2009; Ganapathy et al., 2015; Lerner et al., 2015; Wang et al., 2016; Kahn et al., 2017; Fujihara et al., 2019; Hoefman et al., 2019; Kuenzig et al., 2019; Landfeldt et al., 2019), recognizing some studies did not provide a definition (Kahn et al., 2017; Fujihara et al., 2019). Other studies did not specify care recipients having a chronic condition in their caregiver definitions, but review of these studies showed that most of their care recipients had a chronic condition of some type (Giovannetti et al., 2009; Ganapathy et al., 2015; Wang et al., 2016; Kuenzig et al., 2019; Landfeldt et al., 2019). Our intention was to exclude caregiver participants who were caring for some acute conditions or injuries that were expected to have short-term impact on their work productivity.

### Valuation of Lost Productivity Questionnaire Adaptation

The research team adapted the VOLP to a caregiver version by reviewing existing questionnaires that measured caregiver work productivity losses (iVICQ, CIIQ and WPAI), followed by discussion among the team, including two caregiver partners, two health economists who mainly developed the VOLP patient version, one person-centered outcome expert, one health economist and potential future user of the VOLP, one clinician and potential future user of the VOLP, and two research assistants. In addition to the two caregiver partners and two health economists who mainly developed the VOLP patient version, we believe it is important to include an expert in person-centered outcome measurement and validation as a research team member because productivity loss has been considered and measured not only as a cost component for economic evaluations (Neumann et al., 2016; Yuasa et al., 2021) but also an important person-centered outcome (Hanemoto et al., 2017; Stewart et al., 2018; Zhang and Sun, 2021). The potential future VOLP users to measure productivity loss among caregivers as a person-centered outcome (a clinician investigator) and cost estimates (a health economist) were also included to ensure that VOLP would meet their research purpose.

The team identified an initial set of concepts would be included in the caregiver questionnaire and developed the first draft. This draft was then improved using one-on-one interviews with 7 caregiver study participants, recruited through existing networks of the Family Caregivers of British Columbia (BC), the BC SUPPORT Unit, and the Centre for Health Evaluation and Outcome Sciences via their social media and newsletters, as well as through posters at medical clinics, doctors’ offices, and large social gathering sites, including coffee shops and community centers. The inclusion criteria for caregiver study participants were individuals who were 19 years of age and over; can read and speak English; can provide informed consent; and were caring for a family member or friend with a chronic condition. The interviews focused on work productivity loss concepts, questionnaire flow, and ease of understanding, which was considered as part of feasibility testing of the adapted caregiver VOLP. Once completed, the interview findings were circulated and discussed among the research team (with one caregiver partner) and changes to the draft were made accordingly.

### VOLP Questionnaire Online Conversion

We converted this newly adapted VOLP for caregivers to an online format using the Qualtrics application. We studied existing online questionnaires, with a focus on visual aids and other presentation methods, to improve the user-friendliness of the online caregiver VOLP. We then developed and circulated an initial draft of the online questionnaire among the research team for feedback. The online questionnaire was then tested using one-on-one interviews with 6 study participants (3 caregivers for online caregiver VOLP and 3 patients for online patient VOLP) recruited using the same methods mentioned above, aiming to improve the user-friendliness and test the feasibility. The online patient and caregiver VOLP shared similar questions and same online designs were applied. We therefore combined the feedback from both caregiver and patient study participants. The research team then discussed the interview findings and finalized the online questionnaire. The final online survey including the VOLP caregiver questionnaire can be found in the Supplementary Appendix.

### Feasibility and Validity Testing

To assess the feasibility and validity of the online caregiver VOLP, we administered it to 400 caregivers in Canada, recruited through a market research company (Ipsos), using pre-defined quotas on age, sex, and regions to ensure that our sample had similar distributions to those observed in the survey conducted by Statistics Canada among a general population of caregivers (Sinha, 2012). We also ensured that at least 200 caregivers were currently employed. We focused on two main VOLP outcomes, absenteeism and presenteeism. Two absenteeism outcomes were calculated based on VOLP: (1) the number of days absent from work in the past 3 months due to caregiving (= A); (2) the percent absent work time due to caregiving responsibilities in the past 3 months using the formula: $\frac{A}{A+B}\times 100$ where B = actual number of days worked in the past 3 months. Presenteeism was measured using a percentage time loss while at work in the past 7 days due to caregiving responsibilities, derived from the following formula: $\frac{C-D}{C}\times 100$ where C = total hours they took to complete all work in the past 7 days and D = total hours they would take to complete the same work if they did not have caregiving responsibilities. The 3-month recall period for absenteeism and 7-day recall period for presenteeism were applied in patient VOLP and justified as better recall periods for absenteeism and presenteeism, respectively, in previous studies (Reilly et al., 1993; Revicki et al., 1994; Zhang et al., 2011a). We also looked at absenteeism and presenteeism in volunteer jobs. These values were calculated using the same method, with the exception that for absenteeism, we focused on volunteer hours spent over the past 3 months instead of volunteer days.

We also included the WPAI caregiver version to compare the corresponding main outcomes with VOLP. The WPAI is a commonly used questionnaire to measure work productivity loss due to caregiving responsibilities, using a 7-day recall period. WPAI absenteeism was measured using two methods: (1) the number of hours missed from work in the past 7 days (= E), due to caregiving responsibilities; (2) the percent work time missed due to caregiving responsibilities in the past 7 days using the same formula: $\frac{E}{E+F}\times 100$, where F = actual number of hours worked in the past 7 days. WPAI presenteeism was measured using a percent impairment while working, which was derived from the formula: $\frac{G}{10}\times 100$ where G = the degree that caregiving responsibilities affected productivity while working (measured on a 0 – 10 scale).

In addition, total hours spent on caregiving responsibilities (referred to as caregiving time thereafter) and the severity of the care recipients’ condition reported by the caregiver were used to evaluate known groups/discriminant validity. The caregiving time was determined by the sum of hours spent on 5 categories of caregiving responsibilities: (1) household activities and tasks; (2) personal care; (3) practical support; (4) emotional support; (5) other responsibilities. The question asking for the severity of the care recipients’ condition was adapted from the General Social Survey-Caregiving and Care Receiving developed by Statistics Canada (Government of Canada Sc, 2016). The severity included 3 levels, mild, moderate, and severe. If a participant cared for multiple care recipients, the highest ranking was used.

Feasibility was demonstrated by participant understanding of the VOLP questionnaire during the interviews at the stages of VOLP adaption and online conversion, and the median overall time spent on completing the final online survey.

We examined convergent validity by measuring the correlations between the VOLP and WPAI absenteeism and the correlation between VOLP and WPAI presenteeism because they share some similar constructs. We further compared the correlations between VOLP and WPAI outcomes with their correlations with caregiving time and we expected the former to be larger than the latter. Validation methods used and result interpretations were similar to those used for validating the VOLP patient version, including comparisons of Spearman rank correlations between caregiver VOLP outcomes and WPAI outcomes with those between patient VOLP outcomes and WPAI outcomes found previously (= 0.57 for absenteeism and 0.42 for presenteeism) (Zhang et al., 2011b). Additionally, we compared the correlation values between VOLP and WPAI outcomes and caregiving time to those between WPAI overall work impairment outcome, defined by $\left(\frac{E}{E+F}+\left(1-\frac{E}{E+F}\right)\times \left(\frac{G}{10}\right)\right)\times 100$, and caregiving time in a previous caregiver WPAI validation study (= 0.32) (Giovannetti et al., 2009). We used Spearman rank correlations to accommodate the highly skewed nature of the productivity loss data distributions with excess zero productivity loss (absenteeism and presenteeism) being reported (see Supplementary Appendix Figures 1, 2). We expected the correlation values in this study to be similar to the correlations observed in the previous studies mentioned above. Our term of comparison for the magnitude of the spearman correlations was based on Hinkle et al. (2003): <0.3 represents negligible, 0.3–0.5 low or small, 0.5–0.7 medium, and ≥0.7 high or large correlation.

Wilcoxon tests were used to determine if VOLP and WPAI outcomes varied between two groups determined by recipients’ condition severity (mild and moderate vs. severe) and caregiving time using median as the cut-off. Effect size (Cliff’s Delta, due to highly skewed absenteeism and presenteeism outcomes) was used to determine the discriminative ability between two groups. According to Romano et al., an absolute value of Cliff’s Delta <0.147 represents trivial, 0.147–0.33 small, 0.33–0.474 medium and ≥0.474 large effect (Romano et al., 2006).

### Ethics Statement

This study was approved by the University of British Columbia-Providence Health Care Research Ethics Board (Ethics Certificate No. H19-00329). The interview participants provided their written informed consent to participate in this study. The online survey participants provided their consent online to continue the survey.

## Results

### VOLP Adaptation Based on Team Discussion and Interviews

The first round of interviews for VOLP adaption involved 7 caregiver participants, while the second round of interviews for online conversion involved 3 caregivers and 3 patients. Participant demographics for the interviews covered a diverse sample, including, but not limited to, varying ethnicities (46% European, 31% Asian, 23% other), age groups (31% 30–39 years, 38% 40–49, 23% 50–59, and 8% 60+), and sex assigned at birth (53% female).

At the first stage of the adaptation of VOLP for caregivers, the research team decided to add three major components to the existing VOLP based on the review of previous questionnaires, research team discussion and interview findings (see details below): caregiving time for different caregiving responsibilities, work productivity loss related to volunteer activities, as well as caregivers’ lost job opportunities. Caregiving time for different caregiving responsibilities is captured in the CIIQ. Including caregiving time before asking for the associated absenteeism and presenteeism helps set up the context and scope of what caregiving responsibilities the survey respondents are taking. We adapted caregiving time from four major categories of caregiving responsibilities and their corresponding examples from the CIIQ. These included household activities and tasks; personal care; practical support; and emotional support. However, one additional category was included to reflect additional responsibilities based on our research team discussion. This was defined as “other responsibilities” and included, but not limited to, activities such as attending counseling sessions and planning for their care recipients. In addition to a table including the five categories to capture total caregiving time for each category, we provided an option to use a more detailed table, which participants could use to record their time for each of the examples under each main category. The majority of interview participants preferred this detailed table. They stated that recalling and calculating all of these tasks was already hard enough, and by viewing each example per category, separately, eased their ability to recall their activities in the past week. Please see the supporting quotations in the Supplementary Appendix.

We found that although there were many questionnaires that measured work productivity loss from a paid work perspective, there were few that also looked at the productivity loss on individuals’ volunteer activities. For many caregivers, volunteering is a major component of their life, and needs to be addressed (Burr et al., 2005). Although the questions regarding work productivity loss from a paid work perspective and a volunteer perspective were very similar, a few key changes were made. The most notable change was the units of time used to measure absenteeism. While we used “days” missed over the past 3 months for paid work absenteeism, we used “hours” missed over the past 3 months for volunteer absenteeism. This suggestion was made by the research team during the interview process, to make the participants recall process easier. As many of our participants’ volunteer work did not follow a strict schedule as paid work, many participants had trouble quantifying their volunteer time in terms of days.

For measuring lost job opportunities, we divided the section into three questions: (1) whether they have declined any job offers or opportunities due to caregiving responsibilities; (2) whether any of these job opportunities would have provided additional income; (3) if yes, then to provide either a monthly or yearly estimate on the additional income (in CAD).

We found that interview participants had little issues with work productivity loss concepts, such as absenteeism, presenteeism and employment status changes. Interview participants had the most difficulty in quantifying the time spent on emotional support, as well as the recall period for their caregiving time. During the interview process, participants had issues quantifying their time spent on emotional support. Some participants felt that they spent much more of their time on emotional support than other responsibility categories but most of the time spent on emotional support concurred with the other responsibilities. This makes it difficult to distinguish the time spent on emotional support from other categories to avoid double counting. Although there were no better change suggestions to address this issue from the research team or interview participants, we should be aware of the potential double counting in our post-hoc data analysis.

One common issue the participants encountered related to our choice of recall period, i.e., looking at the past week at the point of taking the questionnaire, as opposed to looking at an average week. They felt that by only looking at the past week, we were not getting a good representation of the time they spent on their caregiving responsibilities over an average week. However, by taking the past week of all participants we would likely get a snapshot and extremes on both ends, a very busy week or a not busy week. One of the main issues with using an average week comes down to how each individual would measure the average week. It would be impossible for us to know or guarantee the consistency in how each individual calculated said average, whereas using the past week is a consistent measure that should not change from person to person. Also, the past week recall period was consistent with that used to measure work productivity loss in VOLP and WPAI.

### Feasibility and Validity Testing

Of the initial 400 online survey participants, we removed 2 individuals who completed the survey in less than 3 min. This value was based on the shortest path required to complete the survey, anything under said limit strongly implied that the participant did not fully read the questionnaire and was less likely to provide meaningful results. We also removed 16 individuals whose reported total hours spent helping their care recipient were deemed too long (assuming the average individual would get 6 h of sleep, anyone whose time reported was over 126 h was removed). This left us with 382 participants. The median overall time spent on completing the online survey was 9.21 min (first quartile: 6.58 – third quartile: 12.23). The completion time for caregivers who were not employed and thus did not answer questions on absenteeism and presenteeism [median = 7.55 min (5.42–11.37)] was shorter than the completion time for caregiver who were employed [median = 9.55 min (7.30–13.23)].

Participant demographics for the feasibility and validity testing survey covered a diverse sample, including, but not limited to, varying ethnicities (59% European, 21% Asian, 4% Aboriginal, 2% Hispanic and 1% African), age groups (20% 25–34 years, 19% 35–44, 32% 45–54 and 29% 55–64), and sex assigned at birth (53% female) (Table 1). About 13% were volunteering, and 61% of care recipients’ chronic conditions were at moderate level. The average total caregiving time in the past 7 days was 33.64 h (standard deviation = 26.80) and the median was 27.00 h (14.12–43.00). The average total caregiving time in the past 7 days, when excluding emotional support, was 28.47 h (standard deviation = 23.44) and the median was 23.00 h (11.00–37.00).

**TABLE 1:** Caregiver participant characteristics.

Variables (*N* = 382)	*N*	%
**Age (years)**		
25–34	75	19.63
35–44	72	18.85
45–54	122	31.94
55–64	110	28.80
**Female**	203	53.14
**Highest level of education completed**		
Primary or high school	73	19.11
College or technical/trade	115	30.10
University	132	34.55
Post-graduate or professional designation	59	15.45
**Ethnicity^a^**		
Aboriginal	15	3.93
African	5	1.31
Hispanic, Latino or Spanish	6	1.57
European	227	59.42
East Asian	55	14.40
South Asian	16	4.19
West Asian	9	2.36
**Caregivers who were doing volunteer work**	51	12.83
**Occupations^b^**		
Management	97	25.39
Finance	29	7.59
Natural and applied science	34	8.90
Health	17	4.45
Education, law, social, community and government services	32	8.38
Art, culture, recreation and sports	9	2.36
Sales and service	40	10.47
Trades and transport	7	1.83
Agriculture and manufacturing	7	1.83
**Severity of care recipients’ chronic conditions**		
Mild	57	14.92
Moderate	234	61.26
Severe	77	20.16
**Health status of caregivers**		
Poor	23	6.02
Fair	61	15.97
Good	138	36.13
Very good	118	30.89
Excellent	41	10.73
**Province of residence**		
Alberta	46	12.04
Atlantic Region^c^	28	7.33
British Columbia	52	13.61
Manitoba	14	3.66
Ontario	163	42.67
Quebec	72	18.85
Saskatchewan	7	1.83

*The count may not add up to 382 because there was a “prefer not to say” option for questions asking participant characteristics or a category having smaller than 5 individuals.*

*^*a*^Our questionnaire allowed multiple choices to be selected for ethnicity.*

*^*b*^Only applicable to caregivers who were employed.*

*^*c*^Includes New Brunswick, Newfoundland and Labrador, Nova Scotia and Prince Edward Island.*

Based on VOLP, of the 277 (73%) participants who were employed either full time, part time or self employed, only 124 reported absence from work in the past 3 months (absenteeism) due to their caregiving responsibilities, with an average of 10.05 absent workdays (median = 4 days) accounting for 20.63% (median = 7.93%) of their work time (Table 2). Of the 232 participants who had worked in the past 7 days, only 81 reported a loss while at work due to their caregiving responsibilities (presenteeism) with an average of 25.36% time loss (median = 20%). Of the 232 participants who had worked in the past 7 days, 155 reported having worked from home. Based on WPAI, the average number of absent work hours was 3.10 h in the past 7 days versus 4.50 absent workdays in the past 3 months from the VOLP. As expected, WPAI provided a much higher presenteeism estimate than VOLP (37.62% versus 8.86%).

**TABLE 2:** VOLP and WPAI outcomes.

Variable	*N*	Mean (SD)	Median (Q1–Q3)
**VOLP outcomes**			
Employed^a^	277		
**Absenteeism due to caregiving responsibilities in the past 3 months**			
Number of absent workdays	277	4.50 (12.84)	0.00 (0.00–4.00)
Number of absent workdays (absent workdays > 0)	124	10.05 (17.70)	4.00 (2.00–7.00)
% work time absent	249	10.28 (20.75)	0.00 (0.00–7.69)
% work time absent (absent workdays > 0)	124	20.63 (25.55)	7.93 (4.51–25.89)
Caregivers who have worked in the past 7 days	232		
Caregiver who worked from home in the past 7 days	155		
**Presenteeism due to caregiving responsibilities in the past 7 days**			
% time loss while working	232	8.86 (16.41)	0.00 (0.00–13.41)
% time loss while working (time loss while working > 0)	81	25.36 (18.81)	20.00 (12.50–33.30)
**WPAI outcomes**			
Caregivers who were working for pay	262		
**Absenteeism due to caregiving responsibilities in the past 7 days**			
Number of absent workhours	262	3.10 (8.89)	0.00 (0.00–2.00)
Number of absent workhours (absent workhours > 0)	90	9.03 (13.32)	5.00 (2.00–8.00)
% work time absent	235	11.29 (23.55)	0.00 (0.00–8.62)
% work time absent (absent workhours > 0)	90	29.47 (30.27)	14.29 (6.25–50.00)
Caregivers whose actual work hour > 0 in the past 7 days	227		
**Presenteeism due to caregiving responsibilities in the past 7 days**			
% impairment while working	227	37.62 (28.74)	40.00 (10.00–60.00)
% impairment while working (impairment while working > 0)	186	45.91 (25.03)	50.00 (20.00–70.00)
**Overall work impairment**	227	40.74 (30.91)	40.00 (10.00–70.00)

*VOLP, the Valuation of Lost Productivity questionnaire; WPAI, the Work Productivity and Activity Impairment questionnaire; SD, standard deviation; Q1, the first quartile; Q3, the third quartile.*

*^*a*^Full time, part time, or self employed.*

Of the 49 participants who were currently volunteering, only 18 reported absence from volunteering in the past 3 months due to their caregiving responsibilities, with an average of 58.14 h (Supplementary Appendix Table 1). Of the 28 participants who had volunteered in the past 7 days, only 9 reported a loss while at volunteer work due to their caregiving responsibilities with an average of 43.83% loss. About 26% (*n* = 98) of the participants had declined job opportunities due to caregiving responsibilities and 68 of them reported the declined job opportunities with associated additional average income of approximately $22,000 CAD per year (Supplementary Appendix Table 2).

Our correlation analyses revealed relatively small correlations between VOLP and WPAI outcomes as expected. The correlation between their absenteeism [*r* = 0.49 (95% confidence interval: 0.37–0.60)] was larger than the presenteeism [*r* = 0.36 (0.24–0.47)] (Table 3). Correlations between VOLP outcomes and total caregiving hours ranged from negligible to small, with a greater correlation for absenteeism [*r* = 0.38 (0.27–0.47)] than presenteeism [*r* = 0.22 (0.10–0.34)]. Due to the potential double counting issue arising from measuring emotional support mentioned above, we repeated the same analysis, removing emotional support from total caregiving hours, observing an increase of 0.01 in correlation values. Correlations between WPAI outcomes and total caregiving hours ranged from negligible to small, with a smaller correlation for absenteeism [*r* = 0.27 (0.15–0.38)] than presenteeism [*r* = 0.35 (0.23–0.46)]. After removing emotional support, the correlation values increased by 0.01 or 0.02. The correlation between WPAI overall work impairment and total caregiving hours was small [*r* = 0.36 (0.23–0.48)].

**TABLE 3:** Spearman correlations between VOLP outcomes, WPAI outcomes and caregiving time.

	VOLP absenteeism in days	VOLP absenteeism in %	VOLP presenteeism
WPAI absenteeism in hours	0.49 (0.37–0.60) (*N* = 259)		
WPAI absenteeism in %		0.49 (0.37–0.61) (*N* = 216)	
WPAI presenteeism			0.36 (0.24–0.47) (*N* = 215)

	**VOLP absenteeism in days** **(*N* = 277)**	**VOLP absenteeism in %** **(*N* = 249)**	**VOLP presenteeism** **(*N* = 232)**

Total caregiving hours	0.38 (0.27–0.47)	0.39 (0.28–0.49)	0.22 (0.10–0.34)
Total caregiving hours excluding emotional support	0.39 (0.29–0.47)	0.40 (0.29–0.50)	0.23 (0.10–0.35)
Total hours spent on emotional support	0.18 (0.04–0.30)	0.18 (0.05–0.30)	0.18 (0.06–0.30)

	**WPAI absenteeism in hours** **(*N* = 262)**	**WPAI absenteeism in %** **(*N* = 235)**	**WPAI presenteeism** **(*N* = 227)**

Total caregiving hours	0.27 (0.15–0.38)	0.27 (0.14–0.40)	0.35 (0.23–0.46)
Total caregiving hours excluding emotional support	0.28 (0.15–0.38)	0.28 (0.16–0.40)	0.37 (0.25–0.50)
Total hours spent on emotional support	0.09 (−0.03 to 0.22)	0.07 (−0.07 to 0.21)	0.13 (−0.01 to 0.26)

*Values presented as Spearman rank correlation and 95% Confidence Interval using Bootstrapped methods with 1,000 iterations.*

*VOLP, the Valuation of Lost Productivity questionnaire; WPAI, the Work Productivity and Activity Impairment questionnaire; VOLP absenteeism in days refers to number of absent workdays due to caregiving responsibilities in the past 3 months; VOLP absenteeism in % refers to % work time absent due to caregiving responsibilities in the past 3 months; WPAI absenteeism in hours refers to number of absent work hours due to caregiving responsibilities in the past 7 days; WPAI absenteeism in % refers to % work time absent due to caregiving responsibilities in the past 7 days.*

Dividing participants into two groups according to the chronic condition severity of their care recipients, the results for VOLP presenteeism and WPAI absenteeism and presenteeism outcomes were not logical with greater loss estimates in mild/moderate status than severe status (Table 4). VOLP absenteeism in % work time absent was statistically significantly larger in caregivers whose care recipients had severe chronic conditions with effect size = 0.20 (i.e., small effect). These results indicated that the VOLP presenteeism and WPAI absenteeism and presenteeism could not discriminate between caregivers whose care recipients had different chronic condition severity levels. The VOLP and WPAI outcomes among caregivers who spent fewer hours on caregiving responsibilities were significantly lower than those among caregivers spending more time. According to the effect size, the VOLP and WPAI could also discriminate between caregivers with less and more caregiving time (small to medium effect size, 0.19 to 0.30 and 0.23 to 0.31, respectively).

**TABLE 4:** VOLP outcomes and WPAI outcomes between two different caregiver groups defined by the condition severity of care recipients and the median of total caregiving hours.

		Condition severity (mild/moderate vs. severe)	Total caregiving hours
VOLP absenteeism in days, median (Q1–Q3)	Better	0.00 (0.00–3.00) *N* = 218	0.00 (0.00–2.00) *N* = 142
	Worse	1.00 (0.00–4.13) *N* = 48	2.00 (0.00–5.00) *N* = 135
	*P*-value	0.17	<0.001
	Effect size	0.12 (−0.04 to 0.27)	0.29 (0.17–0.41)
VOLP absenteeism in %, median (Q1–Q3)	Better	0.00 (0.00–7.69) *N* = 202	0.00 (0.00–3.61) *N* = 125
	Worse	4.76 (0.00–9.89) *N* = 40	4.62 (0.00–14.29) *N* = 124
	*P-*value	0.03	<0.001
	Effect size	0.20 (0.02–0.36)	0.30 (0.17–0.42)
VOLP presenteeism, median (Q1–Q3)	Better	0.00 (0.00–15.42) *N* = 184	0.00 (0.00–3.39) *N* = 124
	Worse	0.00 (0.00–12.50) *N* = 38^a^	0.00 (0.00–25.00) *N* = 108
	*P*-value	0.82	<0.001
	Effect Size	−0.02 (−0.18 to 0.14)	0.19 (0.06–0.31)
WPAI absenteeism in hours, median (Q1–Q3)	Better	0.00 (0.00–2.00) *N* = 206	0.00 (0.00–0.00) *N* = 134
	Worse	0.00 (0.00–1.00) *N* = 46^a^	0.00 (0.00–3.25) *N* = 128
	*P*-value	0.73	<0.001
	Effect size	−0.03 (−0.18 to 0.13)	0.23 (0.11–0.34)
WPAI absenteeism in %, median (Q1–Q3)	Better	0.00 (0.00–9.09) *N* = 186	0.00 (0.00–2.44) *N* = 117
	Worse	0.00 (0.00–7.41) *N* = 41^a^	1.22 (0.00–16.96) *N* = 118
	*P*-value	0.74	<0.001
	Effect size	−0.03 (−0.20 to 0.14)	0.24 (0.11–0.36)
WPAI presenteeism, median (Q1–Q3)	Better	40.00 (10.00–60.00) *N* = 181	20.00 (10.00–50.00) *N* = 115
	Worse	30.00 (10.00–55.00) *N* = 39	50.00 (20.00–70.00) *N* = 112
	*P*-value	0.76	<0.001
	Effect size	−0.03 (−0.22 to 0.16)	0.31 (0.16–0.44)
Total caregiving hours, median (Q1–Q3)	Better	25.00 (14.00–42.00) *N* = 291	N/A
	Worse	32.00 (16.00–44.00) *N* = 77	N/A
	*P*-value	0.16	
	Effect size	0.11 (−0.04 to 0.25)	N/A

*Better status was defined as mild or moderate or ≤ median (= 27 h) of caregiving hours, and worse status was defined as severe or > median of caregiving hours. VOLP, the Valuation of Lost Productivity questionnaire; WPAI, the Work Productivity and Activity Impairment questionnaire; Q1, the first quartile; Q3, the third quartile; N/A, not applicable.*

*^*a*^Indicates *N* for non-zero values were ≤15.*

## Discussion

By applying mixed methods and caregiver-oriented research, we adapted the VOLP patient version for measuring work productivity loss among caregivers. The feasibility was supported by participant understanding of the caregiver VOLP shown in the interviews and the reasonable time spent on completing the final online survey. Our validity testing showed that the correlations between VOLP outcomes and WPAI outcomes were small and the correlation between the VOLP and WPAI presenteeism was weaker than the correlations between their absenteeism outcomes. This weaker correlation reflects differences in how presenteeism was measured: the VOLP using direct time measurement method vs. the WPAI using a 0–10 scale. When assessing presenteeism of patients with osteoarthritis or rheumatoid arthritis, a previous study that compared the 0–10 scale of the WPAI to direct hour estimating method of the Health and Labor Questionnaire found the correlation to be 0.37, which is similar to our results produced (Zhang et al., 2010). The correlations found in our study were slightly smaller than the previous validation results for the VOLP patient version, in which the correlation between VOLP absenteeism and WPAI absenteeism was 0.57 (vs. 0.49 in this study) and the correlation between presenteeism outcomes was 0.42 (vs. 0.36) (Zhang et al., 2011b).

We also found the correlations between the VOLP outcomes and WPAI outcomes were larger than those between the VOLP and caregiving time and condition severity of care recipients. This suggested that VOLP outcomes share more similar constructs to WPAI outcomes than caregiving time and condition severity of care recipients. We noted larger correlations between VOLP absenteeism and caregiving time than WPAI absenteeism. Similarly, VOLP absenteeism had a larger effect size than WPAI absenteeism when discriminating groups with lower and higher caregiving time. These may be attributed to the different recall periods used, with the longer 3-month period being used by the VOLP compared to the shorter 7-day period of the WPAI. Revicki et al. (1994) demonstrated that reporting absent workdays over 3 months was as accurate as those of a month, and the extended time period may itself lead to more stable estimates. This might also reflect the recall issue for caregiving time raised by our interview participants and some of the interview participants might report their caregiving time in an average week in a longer time period instead of the past week as instructed. The weaker correlation seen between VOLP presenteeism and caregiving time and smaller effect size than WPAI presenteeism may again reflect their different constructs used to measure presenteeism. Compared to the findings from the previous caregiver WPAI validation study, the correlation between WPAI overall work impairment and caregiving time in our study was slightly larger (0.36 vs. 0.32) (Giovannetti et al., 2009).

When comparing VOLP and WPAI outcomes and caregiving time by care recipients’ condition severity, we noted more illogical trends than logical. The instances of logical trends were seen when comparing VOLP absenteeism and caregiving time. Only VOLP absenteeism as a percent work time absent and caregiving time revealed statistically significant differences between mild/moderate and severe groups. The large number of illogical trends seen, and lack of significant results might be due to the small sample sizes among the subgroups and the highly skewed outcome data with excess zeros. This may also be due to use of a single question, adapted from the General Social Survey-Caregiving and Care Receiving developed by Statistics Canada (Government of Canada Sc, 2016), that involves reliance on caregivers as proxies to assess the severity of their care recipients’ conditions. Future studies that use larger sample sizes and link to care recipient self-reported disease severity can investigate this further to determine whether the VOLP and WPAI could discriminate caregivers based on their care recipients’ disease severity.

We observed the time spent on emotional support accounting for 15.4% of the total caregiving time. Although our interview participants realized to avoid double counting the time for each category of caregiving responsibilities, they mentioned that it could be challenging to do so for emotional support. Thus, we suggest researchers conducting sensitivity analyses by including and excluding emotional support in their future studies. When testing the convergent validity of VOLP, we found that including and excluding emotional support had minimal effect on our correlation values.

The patient VOLP was developed based on economic theory and applies different measurement methods including a different recall period for absenteeism (3 months vs. 1 week for WPAI and CIIQ) and a direct time measurement approach for presenteeism compared to 0–10 scale used by WPAI and CIIQ and multidimensional measurement by WLQ (Zhang et al., 2011a, 2012). The 3-month recall period for absenteeism has been chosen based on the previous evidence on its accuracy on reporting absent workdays and as it is a common follow-up time point in clinical trials (Revicki et al., 1994; Zhang et al., 2011a). As mentioned above, different presenteeism measurement methods provide widely varied estimates (Zhang et al., 2010). The direct time measurement for presenteeism used by VOLP provides a direct work time loss estimate that could be converted to productivity loss in monetary value. In addition, the VOLP can be used to measure productivity loss for volunteer work, which is a major component of caregivers’ life.

Furthermore, we adapted questions regarding the time spent on different caregiving responsibilities based on CIIQ and examined the questions through interviews with caregivers. We started asking survey participants the questions regarding the time spent on caregiving responsibilities to give them a better understanding of the concepts that would be utilized to answer the related productivity loss questions. CIIQ first asks for absenteeism and presenteeism “due to your relative’s disease/condition” by providing some examples of caregiving responsibilities and then asks questions on the time spent on each category of caregiving responsibilities. This way could lead to inconsistencies among these questions. We did not change the order of items in the VOLP questionnaire. The questions within VOLP have been set up in a logic order so that survey participants who are not eligible for certain questions will skip. For example, the question regarding employment status will determine who is eligible for absenteeism questions or not. After absenteeism questions, those who have worked in the past 7 days will be eligible for presenteeism questions. However, we did not randomize the order of VOLP and WPAI, which might lead to order effects bias.

Our study had several additional limitations. Our limited sample sizes made it difficult to produce meaningful statistical results by different caregiver groups, e.g., by care recipients’ condition severity. Additionally, many participants reported zero-values especially for VOLP presenteeism or WPAI absenteeism, which requires even larger sample sizes and special considerations on analysis methods in future studies.

Another limitation in our study arose from the period the study was undertaken, as our feasibility and validity testing survey was launched during the height of the COVID-19 pandemic (between May 15, 2020 to June 2, 2020). During that period, social distancing and gathering restrictions were implemented across Canada, which had significant impact on health care service access (related to caregiving time) and work arrangements (related to caregivers’ work productivity loss). In response to this, we included a question about whether caregiver responders were working from home and found a two-third of the participants who had worked in the past 7 days, had worked from home. Our findings could be valid only under this situation with more people working from home. However, our findings will still be relevant in a post-COVID-19 caregiving environment if more caregivers can work from home. Furthermore, as the survey was launched in the first few months of dealing with the COVID-19 outbreak, caregivers might have had a hard time adapting their caregiving and working life, which could have been reflected in their reported caregiving time and productivity loss results. However, this would not affect our validation results, as it would have been reflected in both the VOLP and WPAI questionnaires.

In summary, the study provides evidence of feasibility and preliminary validity evidence of the adapted VOLP caregiver questionnaire in measuring productivity losses due to caregiving responsibilities, when compared with the results for WPAI and the results from the previous VOLP validation study among patients. In addition to absenteeism and presenteeism for caregivers’ paid employment, researchers could measure their caregiving time, absenteeism and presenteeism for volunteer work, and lost opportunities. Special considerations should be given to the recall period for caregiving time, the potential double counting issue by including emotional support, and the appropriate sample size due to the highly skewed data with excess zeros.

## Data Availability Statement

The raw data supporting the conclusions of this article will be made available from the corresponding author upon reasonable request. The data are not publicly available due to ethical restrictions.

## Ethics Statement

The studies involving human participants were reviewed and approved by The University of British Columbia-Providence Health Care Research Ethics Board (Ethics Certificate No. H19-00329). The interview participants provided their written informed consent to participate in this study. The online survey participants provided their consent online to continue the survey.

## Author Contributions

WZ conceived and designed the study. WZ, CL, RS, AP, and AHA were the investigators in funding application. AG and JS were the primary interviewers and analyzed the qualitative data. AG primarily worked on the online conversion using the Qualtrics application, and performed the data analysis of the survey. WZ and RS provided guidance on the data analysis. AG and WZ drafted the manuscript. All authors were involved in developing the adapted caregiver questionnaire and finalizing the questionnaire in both paper version and online version, and involved in editing and reviewing the manuscript and have given approval to the final manuscript.

## Conflict of Interest

KP is a freelancer and collaborates with Section 2 Productions. The remaining authors declare that the research was conducted in the absence of any commercial or financial relationships that could be construed as a potential conflict of interest.

## Publisher’s Note

All claims expressed in this article are solely those of the authors and do not necessarily represent those of their affiliated organizations, or those of the publisher, the editors and the reviewers. Any product that may be evaluated in this article, or claim that may be made by its manufacturer, is not guaranteed or endorsed by the publisher.
