# Supplementary material for: Valuation of Lost Productivity in Caregivers: A Validation Study
Source: Front Psychol. 2021 Aug 27;12:727871. doi: 10.3389/fpsyg.2021.727871 (PMC8432932; doi:10.3389/fpsyg.2021.727871)
Supplement: Supplementary file 1 [file Data_Sheet_1.docx]

**Appendix:**

**Other Questionnaire Details:**

The Work Productivity and Activity Impairment (WPAI) is an easily generalizable questionnaire able to cover many diseases, occupations and caregiving. Many studies have evaluated its reliability and validity among patients (1,2) . Data extracted from the WPAI can be used to measure absenteeism, presenteeism and working status. Its scoring method is intuitive and has low respondent burden in part due to having a low number of questions (6 questions at maximum) (3). However, the WPAI is not without its limitations. The method used to measure presenteeism relies on a 0-10 scale, which can lead to larger time loss estimates of presenteeism when compared to direct time measurement methods and the estimate from the Work Limitations Questionnaire (WLQ) mentioned below (4). These larger time loss estimates are more likely to lead to an overestimation in the actual productivity loss due to presenteeism because it might capture the quality of life and psychosocial impacts as well (4,5).

The WLQ is another widely used measurement tool when assessing work productivity while at work (presenteeism), with repeated evidence of positive construct validity (3,6–8). It is a multi-dimensional questionnaire including 25 items and thus the breadth of potential work limitations examined is high and should have strong relevance across many job types and health conditions. A Productivity Loss Index can be derived from WLQ, and further used to estimate the percentage of productivity loss and then convert it into time loss. However, it increases the burden of response. Furthermore, the WLQ does not include questions for absenteeism and employment status. The WLQ uses a 2 week recall period while many of the above questionnaires use a 1 week recall period because of the concern of recall bias regarding presenteeism (3,6–8).

The Caregiver Indirect and Informal Care Cost Assessment Questionnaire (CIIQ) is a newer questionnaire (developed in 2018) and the authors stated that it is the first instrument to measure, value and estimate productivity losses and overall care costs for caregivers (9). The CIIQ was developed based on iMTA Valuation of Informal Care Questionnaire (iVICQ) and thus the two questionnaires share similar questions (9,10). Similar to the WPAI, the CIIQ uses a 0-10 scale when measuring presenteeism, and thus more likely to overestimate the actual productivity losses due to presenteeism when compared to a direct time measurement method. Additionally, the CIIQ has not been tested, applied or validated among a population of caregivers.

References:

1. Reilly MC, Zbrozek AS, Dukes EM. The Validity and Reproducibility of a Work Productivity and Activity Impairment Instrument: PharmacoEconomics. 1993 Nov;4(5):353–65.

2. Reilly Associates. WPAI Studies [Internet]. [cited 2017 Aug 8]. Available from: http://www.reillyassociates.net/WPAI_References.html

3. Tang K, Beaton DE, Boonen A, Gignac MAM, Bombardier C. Measures of work disability and productivity: Rheumatoid Arthritis Specific Work Productivity Survey (WPS-RA), Workplace Activity Limitations Scale (WALS), Work Instability Scale for Rheumatoid Arthritis (RA-WIS), Work Limitations Questionnaire (WLQ), and Work Productivity and Activity Impairment Questionnaire (WPAI). Arthritis Care Res. 2011 Nov;63(S11):S337–49.

4. Zhang W, Gignac MAM, Beaton D, Tang K, Anis AH. Productivity Loss Due to Presenteeism Among Patients with Arthritis: Estimates from 4 Instruments. J Rheumatol. 2010 Sep;37(9):1805–14.

5. Zhang W, Bansback N, Anis AH. Measuring and valuing productivity loss due to poor health: A critical review. Social Science & Medicine. 2011 Jan;72(2):185–92.

6. Lerner D, Amick BC, Lee JC, Rooney T, Rogers WH, Chang H, et al. Relationship of Employee-Reported Work Limitations to Work Productivity: Medical Care. 2003 May;41(5):649–59.

7. Lerner D, Amick BC, Rogers WH, Malspeis S, Bungay K, Cynn D. The Work Limitations Questionnaire: Medical Care. 2001 Jan;39(1):72–85.

8. Lerner D, Parsons SK, Chang H, Visco ZL, Pawlecki JB. The Reliability and Validity of the Caregiver Work Limitations Questionnaire. Journal of Occupational & Environmental Medicine. 2015 Jan;57(1):22–31.

9. Landfeldt E, Zethraeus N, Lindgren P. Standardized Questionnaire for the Measurement, Valuation, and Estimation of Costs of Informal Care Based on the Opportunity Cost and Proxy Good Method. Appl Health Econ Health Policy. 2019 Feb;17(1):15–24.

10. Hoefman R, van Exel N, Brouwer W. iVICQ: iMTA Valuation of Informal Care Questionnaire, Version 1.1 [Internet]. [cited 2019 Jan 10]. Available from: https://www.imta.nl/assets/uploads/2018/01/iVICQ_UK_version_1.1.pdf

**Supporting Qualitative Evidence:**

| **Section, page number** | **Section from Manuscript** | **Supporting Quotes** |
| --- | --- | --- |
| Results, pp. 5 | “The majority of interview participants preferred this detailed table. They stated that recalling and calculating all of these tasks was already hard enough, and by viewing each example per category, separately, eased their ability to recall their activities in the past week.” | Participant 01: “Actually, anything that can break it down is helpful (referring to detailed table)… It is, yeah, because you sort of get lost in just the doing and the habits, and you kind of forget all of the things that you do. Because even when I was on respite care last week, all the memos I was doing and instructions, it really helped sort of bring that awareness of the detailing of the task.” |
|  |  | Participant 06: “I think I would prefer this table (indicating detailed table)… To work through that because I had to do it anyways, like, before. Like, this is the first time I’ve seen it (referring to the detailed table) because I didn’t read the whole questionnaire at the time… I already had a spreadsheet that looks like this (referring to the detailed table)”  Interviewer: “So the overall thought process here is that you prefer this one with the more details?”  Participant 06: “Yes.” |
|  |  | Interviewer: “If you don’t mind, if you want to look through these two tables and see which one you would prefer and what’s easier and more conceptual for you.”  Participant 07: “I’m thinking the alternate table (detailed table), the one I’m looking at on page 10… Makes more sense to me… Yeah, I have filled something like this out before… I was at an orthopaedic surgeon’s office, and I remember seeing a form that was very similar to this… So this makes sense to me.” |
| Results, pp. 6 | “For measuring lost job opportunities, we divided the section into three questions: 1) whether they have declined any job offers or opportunities due to caregiving responsibilities; 2) whether any of these job opportunities would have provided additional income; 3) if yes, then to provide either a monthly or yearly estimate on the additional income (in CAD).” The initial question combined parts 2) and 3), however this was found to invoke more stress in participants and was easier to answer when each part was asked separately. | Participant 01: “Wow that’s a stressful question to ask because it’s sort of you are realizing, you know, your worth, and maybe what you’re not getting paid sort of thing.” |
|  |  | Participant 06: “Going in the vein of answering across all time, early on in my caregiving responsibilities, I did have to withdraw an application form, I mean, whether I would have gotten it or not, but trusting that I would have been eligible for this, um, how do I even quantify its dollar value back then… I’m going back almost 10 years ago, so I think I’m having a tough time thinking in that term.” |
| Results, pp. 6 | “Some participants felt that they spent much more of their time on emotional support than other responsibility categories but most of the time spent on emotional support concurred with the other responsibilities. This makes it difficult to distinguish the time spent on emotional support from other categories to avoid double counting.” | Participant 01: “Emotional support, for example, helping to cope with pain, disability and discomfort, anxiety and worry for your care recipient. That’s an interesting question… Hard to quantify that one.” |
|  |  | Participant 02: “I think emotional support, I don’t know. It’s kind of always there, so I’m not sure that I would feel able to quantify this… The emotional support, you know, whenever he needs it, and I don’t know that it’s really anything that I think about. I mean, for me, I don’t think about it, but very often, he’ll say, “Thanks just for being here.” As far as anxiety and worry, it really is as long as, and even sometimes when I’m sleeping, but certainly while I’m awake. If I’m not concentrating on other things, I’m probably worried about how he’s doing… Maybe other people can quantify it, but I’m gonna, I’m gonna kind of skip it and not put a number down.” |
|  |  | Participant 04: “I mean, I read this (referring to emotional support question), and it’s like, that’s 24/7… You know, I mean, for the last 11 months, I’ve been with my mom 23 hours out of the day… I’d put 24 hours a day.” |
| Results, pp. 6 | “One common issue the participants encountered related to our choice of recall period, i.e., looking at the past week at the point of taking the questionnaire, as opposed to looking at an average week. They felt that by only looking at the past week, we were not getting a good representation of the time they spent on their caregiving responsibilities over an average week.” | Participant 01: “Yeah, I tried to average it out (referring to time spent on caregiving)… If I did the survey based on an exceptional week, it would look like, you know, I was living in emergency all the time… And I guess, too, because this, for me, has been a very long period of time that I’ve been doing it, essentially since 2010, it’s been peaks and valleys… I have a lot of experiences to sort of weed out… So I’m trying to, you know, not think about four years ago when things were in crisis, because right now, we’re not in crisis.”  Interviewer: “Would it be correct to say that you sort of interpreted ‘in the past seven days’ to being an average?”  Participant 01: “Yeah.” |
|  |  | Participant 02: “Again, I kinda feel, especially if you’re trying to research the overall effect of caregiving responsibilities on the caregiver’s work productivity, I would feel like if I’m answering this and I say, ‘Okay. Specifically the last seven days, no’, but I feel like it’s doing me and all other caregivers a great disservice because, on an average week, then I’m probably more around, like, a six in terms of how it affects my performance at work.” |

**Final Online Survey Link and Instructions**

To access an online survey (Consent section is excluded), please use the following link: <https://ubc.ca1.qualtrics.com/jfe/preview/SV_0NRqsQseDmdUQh8?Q_CHL=preview&Q_SurveyVersionID=current>

When accessing the link, one may change the method of going through the questionnaire via the Tools options on the top right of the page. By selecting ‘Ignore validation’ the user will be able to move through the questionnaire without answering all questions. Please note that certain questions will only appear if specific answers are chosen in previous questions (e.g., all questions related to employment, absenteeism and presenteeism will only appear if the participants answer in question 7 indicates they are employed and all the following questions).

**Appendix Table 1. VOLP volunteering outcomes**

| **Variable** | **N** | **Mean (SD)** | **Median (Q1-Q3)** |
| --- | --- | --- | --- |
| Volunteering | 49 |  |  |
| Absenteeism due to caregiving responsibilities in the past 3 moths |  |  |  |
| Number of absent volunteer hours | 49 | 21.36 (84.25) | 0 (0-7.0) |
| Number of absent volunteer hours (absent volunteer hours >0) | 18 | 58.14 (133.33) | 11 (5.5-36.25) |
| % volunteer time absent | 49 | 17.49 (26.48) | 0 (0-45.83) |
| % volunteer time absent (absent volunteer hours >0) | 18 | 44.70 (23.84) | 51.19 (30.39-65.54) |
| Caregivers who have volunteered in the past 7 days | 28 |  |  |
| Presenteeism due to caregiving responsibilities in the past 7 days |  |  |  |
| % time loss while volunteering | 28 | 14.09 (24.64) | 0 (0-23.33) |
| % time loss while volunteering (time loss >0) | 9 | 43.83 (24.14) | 40 (33.33-47.83) |

VOLP: the Valuation of Lost Productivity questionnaire; SD: standard deviation; Q1: the first quartile; Q3: the third quartile

**Appendix Table 2. Lost job opportunities**

|  | **N** | **Yes** | **No** |
| --- | --- | --- | --- |
| Declined any job opportunities due to caregiving responsibilities | 382 | 98 (26%) | 284 (74%) |
| Would declined job opportunities have provided additional income | 98 | 65 (66%) | 23 (23%) |
|  | **N** | **Mean (SD)** | **Median (Q1-Q3)** |
| Estimate of additional monthly income (in CAD) | 45 | 1755 (3718.18) | 1000 (450 – 2000) |
| Estimate of additional yearly income (in CAD) | 20 | 22775 (15889.81) | 20000 (12000 – 25000) |

**Appendix Figure 1. Boxplots of VOLP Outcomes**


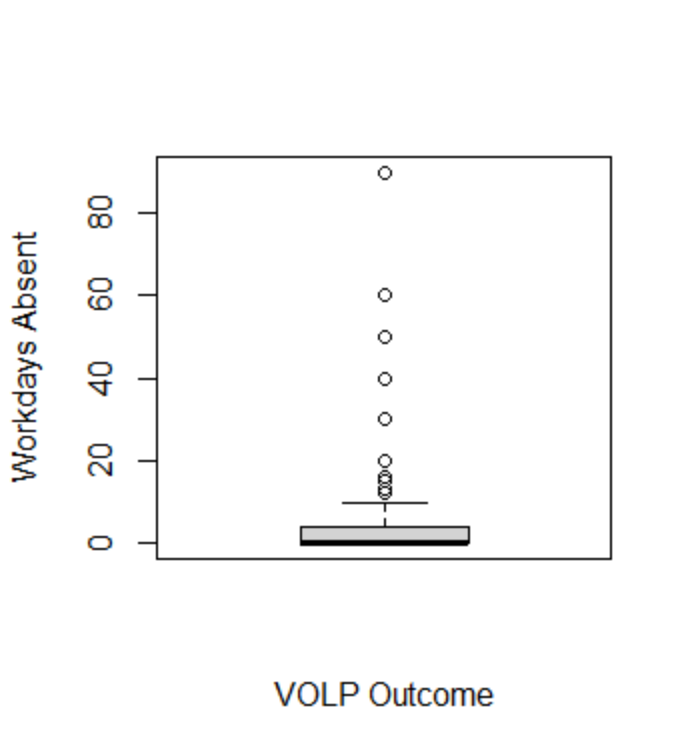

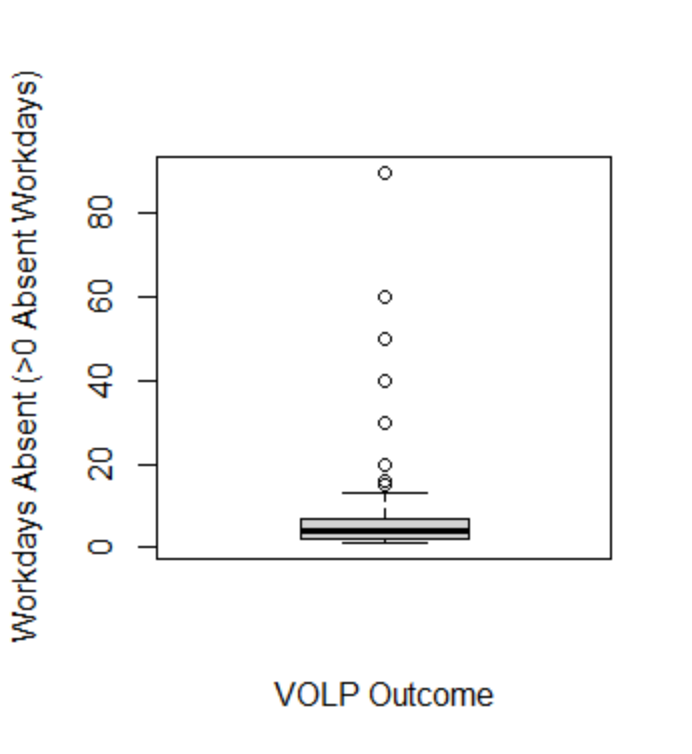


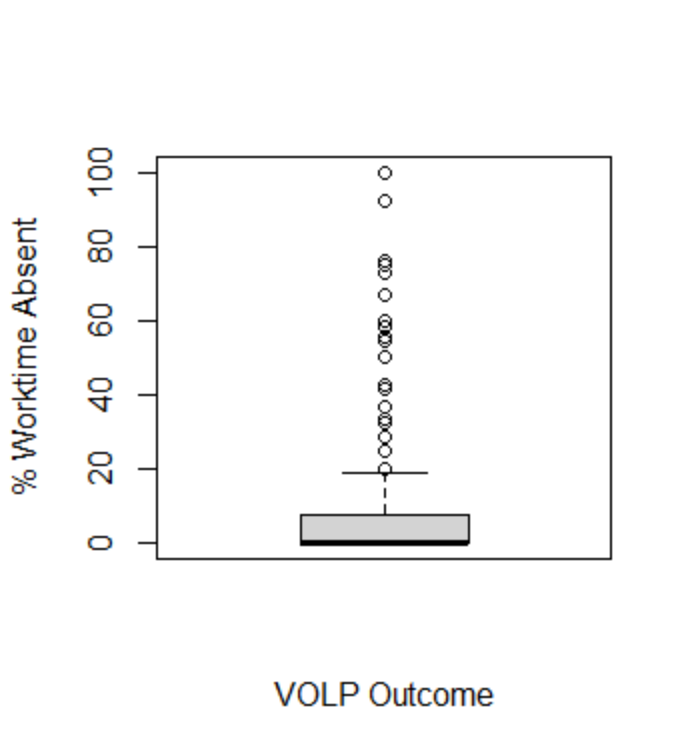

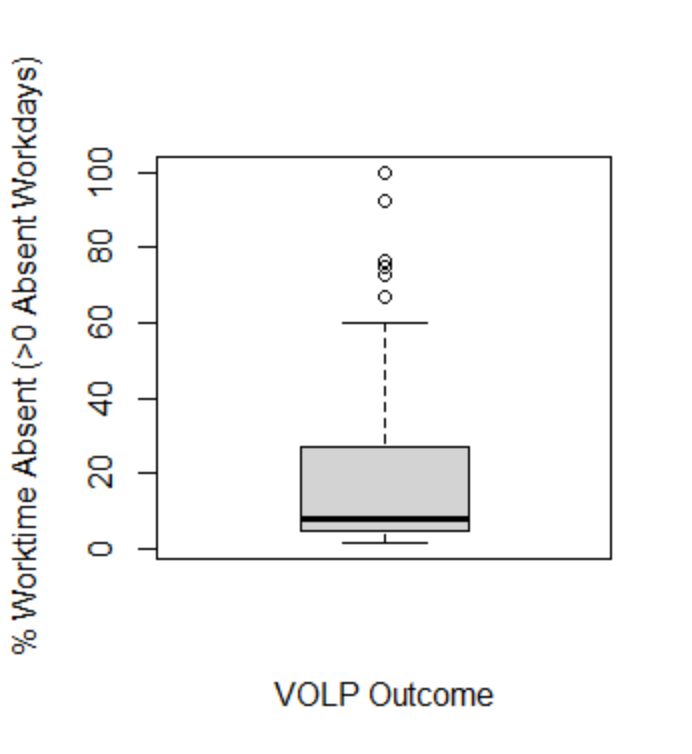


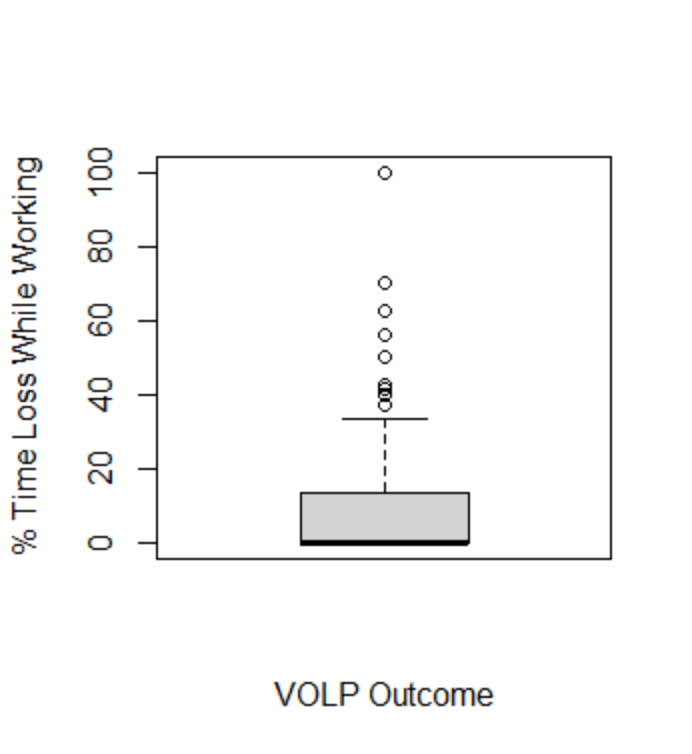

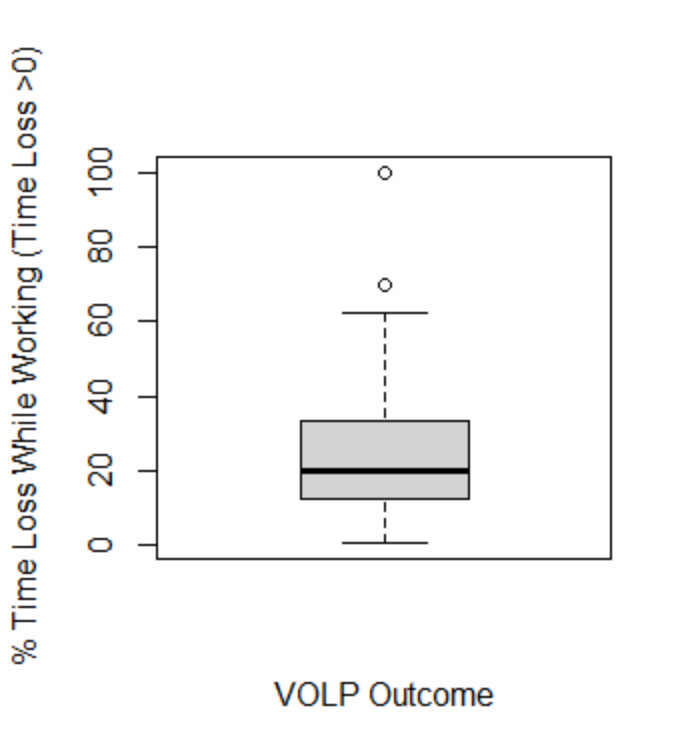


The boxplots depicted in the figure represent the plotted results shown in Table 2 of the main manuscript, depicting VOLP outcomes. The N values for each boxplot are the same as those provided in Table 2. Upon inspection of the boxplots, we can see the highly skewed nature of the productivity loss data (both absenteeism and presenteeism), with the majority of the data points hovering towards 0.

**Appendix Figure 2. Boxplots of WPAI Outcomes**


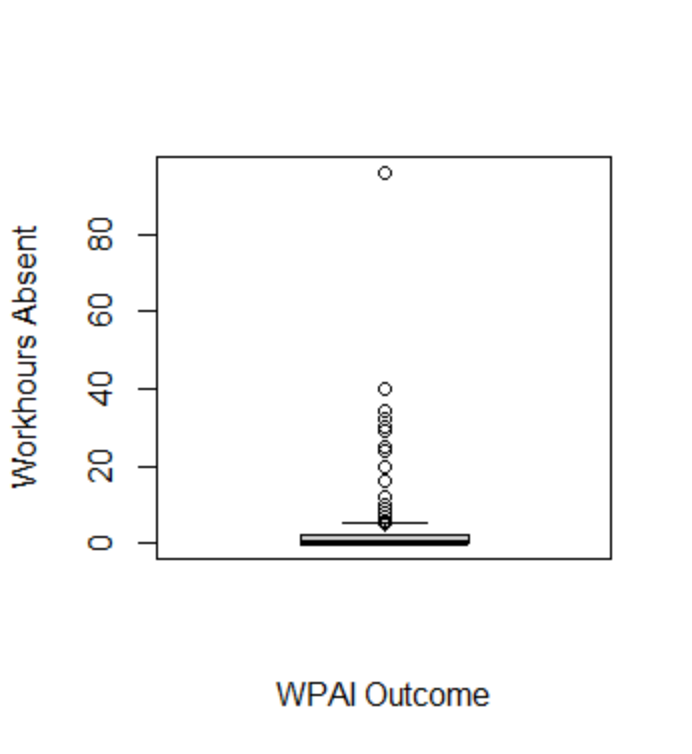

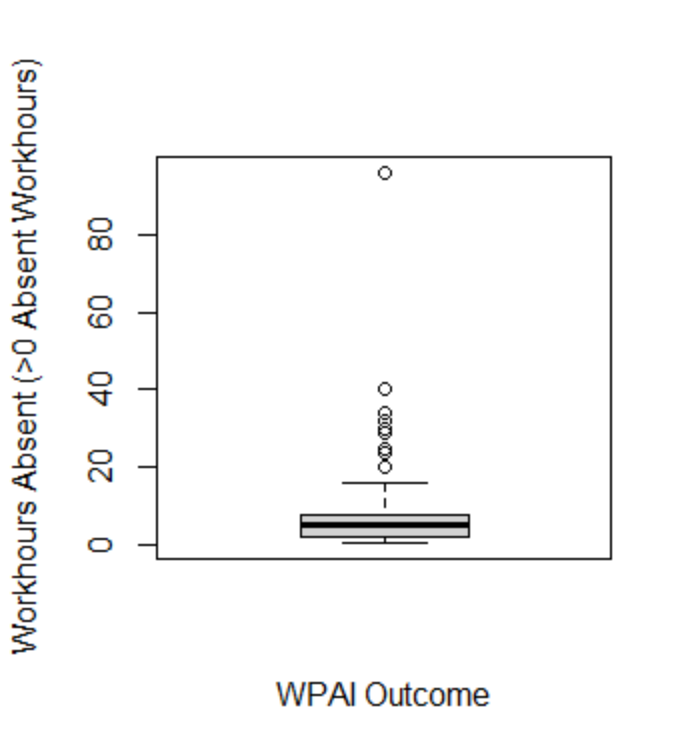


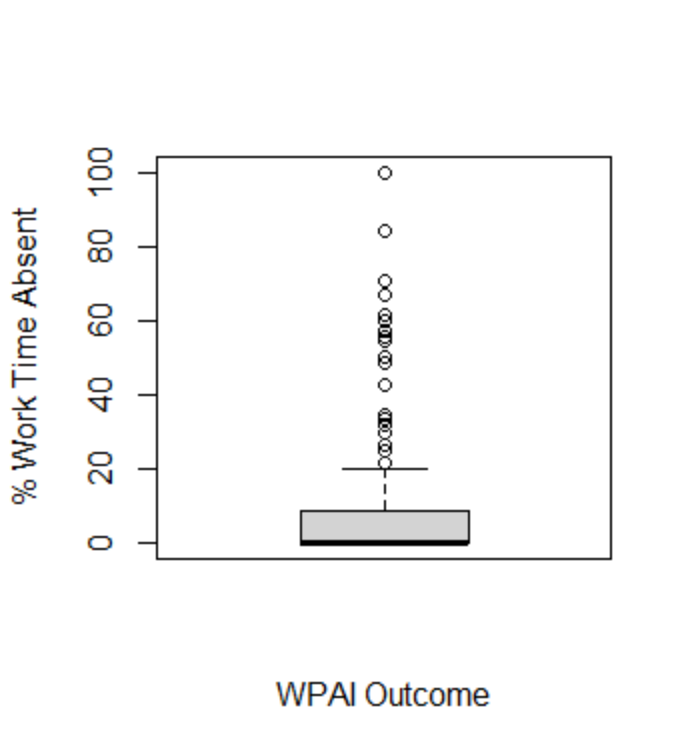

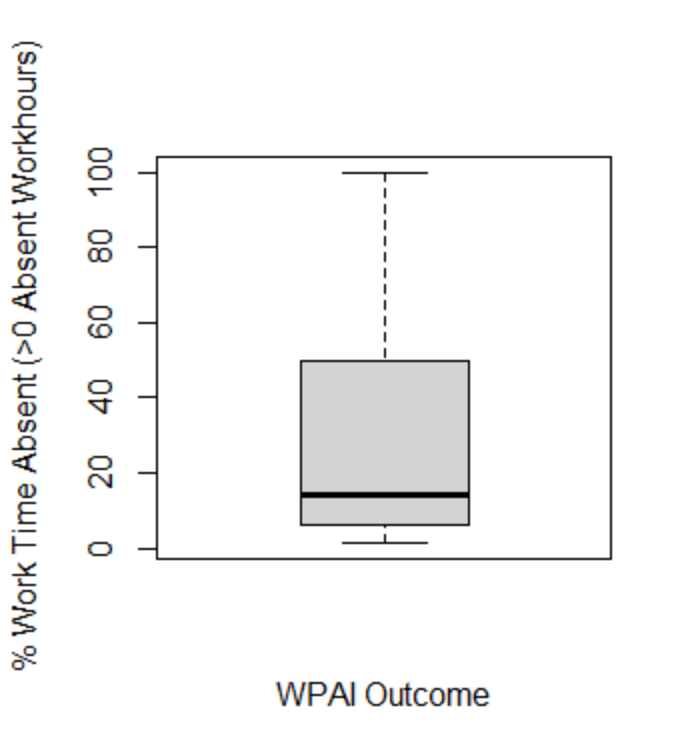


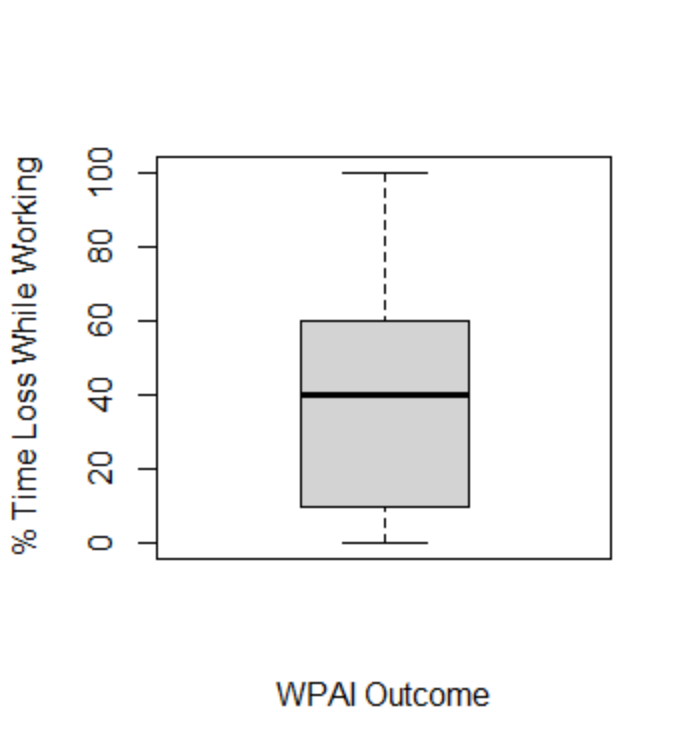

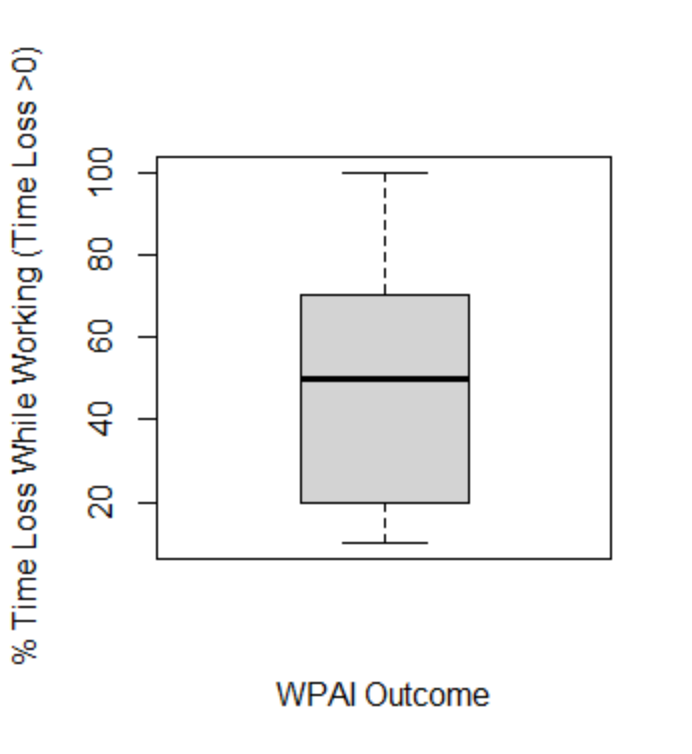


The boxplots depicted in the figure represent the plotted results shown in Table 2 of the main manuscript, depicting WPAI outcomes. The N values for each boxplot are the same as those provided in Table 2. Upon inspection of the first 4 boxplots, we can see the highly skewed nature of the productivity loss data (for absenteeism), with the majority of the data points hovering towards 0. Although this skewdness is not presented in the boxplots depicting % Time Loss While Working (presenteeism), the Shapiro-Wilks test for normality does confirm that the data is not normally distributed.
